# Simulation of human gait with body weight support: benchmarking models and unloading strategies

**DOI:** 10.1186/s12984-020-00697-z

**Published:** 2020-06-25

**Authors:** Salil Apte, Michiel Plooij, Heike Vallery

**Affiliations:** 1 Department of Biomechanical Engineering, Delft University of Technology, Mekelweg 2, Delft, 2628 CD Netherlands; 2 Motek Medical BV, Hogehilweg 18C, Amsterdam, 1101 CD Netherlands; 3 Laboratory of Movement Analysis and Measurement (LMAM), École Polytechnique Fédérale de Lausanne, Station 9, Lausanne, CH-1015 Switzerland

**Keywords:** Body weight support, Gait models, Human locomotion, Gait rehabilitation

## Abstract

**Background:**

Gait training with partial body weight support (BWS) has become an established rehabilitation technique. Besides passive unloading mechanisms such as springs or counterweights, also active systems that allow rendering constant or modulated vertical forces have been proposed. However, only pilot studies have been conducted to compare different unloading or modulation strategies, and conducting experimental studies is costly and time-consuming. Simulation models that predict the influence of unloading force on human walking may help select the most promising candidates for further evaluation. However, the reliability of simulation results depends on the chosen gait model. The purpose of this paper is two-fold: First, using human experimental data, we evaluate the accuracy of some of the most prevalent walking models in replicating human walking under the influence of Constant-Force BWS: The Simplest Walking model (SW), the Spring-Loaded Inverted Pendulum model (SLIP) and the Muscle-Reflex (MR) gait model. Second, three realizations of BWS, based on Constant-Force (CF), Counterweight (CW) and Tuned-Spring (TS) approaches, are compared to each other in terms of their influence on gait parameters.

**Methods:**

We conducted simulations in Matlab/Simulink to model the behaviour of each gait model under all three BWS conditions. Nine simulations were undertaken in total and gait parameter response was analysed in each case. Root mean square error (mrmse) w.r.t human data was used to compare the accuracy of gait models. The metrics of interest were spatiotemporal parameters and the vertical ground reaction forces. To scrutinize the BWS strategies, loss of dynamic similarity was calculated in terms of root mean square difference in gait dynamics (*Δ*gd) with respect to the reference gait under zero unloading. The gait dynamics were characterized by a dimensionless number Modela-w.

**Results:**

SLIP model showed the lowest mrmse for 6 out of 8 gait parameters and for 1 other, the mrmse value were comparable to the MR model; SW model had the highest mrmse. Out of three BWS strategies, Tuned-Spring strategies led to the lowest *Δ*gd values.

**Conclusions:**

The results of this work demonstrate the usefulness of gait models for BWS simulation and suggest the SLIP model to be more suitable for BWS simulations than the Simplest Walker and the Muscle-reflex models. Further, the Tuned-Spring approach appears to cause less distortions to the gait pattern than the more established Counterweight and Constant-Force approaches and merits experimental verification.

## Introduction

Body weight supported training (BWST) is a common gait rehabilitation technique for individuals suffering from neurological impairment due to stroke, spinal cord injury, Parkinson’s disease, etc. During BWST, a certain amount of the user’s body weight is supported by a suspension system, typically through a harness [1]. Thereby, body weight support (BWS) systems allow therapists to provide gait rehabilitation training, without the need of providing complete physical assistance [2]. After undergoing BWST, individuals with neurological impairments have shown improvement in balance, motor function and overall locomotion [3–8]. In addition to these benefits, BWST can lead to improved psychological well-being, enhanced muscle mass and better cardiovascular health [9].

A BWS system is typically composed of an apparatus which provides the user with an unloading force when walking overground or on a treadmill [10, 11]. The main purpose of providing a constant unloading force is to partially reduce gravity. The notion that constant force is the best solution for partial BWS has been dominating the field of BWS systems [12], and led to complex mechanical designs such as the Lokolift [10], the Zero-G [13], etc. These devices use active control in order to render a constant force. Note that this is still different from actual simulated gravity because the load is applied only to the upper body (distributed via the harness), and not in a distributed way on each single body segment [14]. Accurate investigations for swing phase therefore generally require set-ups similar to a parabolic space flight [15, 16], which are inconvenient to reproduce.

Passive, and more low-cost BWS realizations for providing constant unloading force typically constitute the use of an appropriately heavy counterweight or an elastic element such as a spring with specific pretension. While these devices provide constant unloading force in static conditions, the vertical movement of the center of mass (COM) of the user during locomotion leads to a vertical motion of the counterweight or the end-point of the elastic element. This results in the deviation of the unloading force from the set (constant) magnitude and thus these device are generally considered inferior to actuated, closed-loop controlled systems [10]. However, there may still be unexploited potential in such passive realizations. Particularly, it could be possible that a simple elastic support may even bring gait dynamics closer to unsupported gait than an actively rendered constant force, following the hypothesis stated originally in [17].

One way to predict the efficacy of existing and new BWS designs and modulation strategies is by simulating their influence on locomotion of existing gait models. This can improve the efficiency of the design process by speeding up the iteration steps and reducing or postponing the need for hardware prototypes and experiments with human subjects. Examples of such an approach are the studies by Glauser et al., Ma et al. and Lu et al. [18–20]. These examples, however, show that there is a wide range of gait models currently being used for such a simulation and they range from the simplest (mass-spring-damper system) to the most complex musculoskeletal models.

**Fig. 1:**
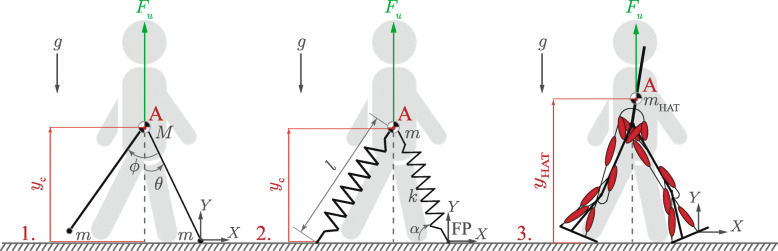
The three gait models considered in this paper: (1) Simplest Walking (SW) model [21] where *M* is the mass of the body, *m* of each foot and *m* is assumed to be negligible as compared to *M*, *θ* is the stance leg angle w.r.t. to vertical, *y*_*c*_ is the vertical position of the centre of mass and *ϕ* is the swing leg angle w.r.t to the stance leg. Details of the actuation principles from [30] are not shown here. (2) Spring-loaded inverted pendulum (SLIP) model [22] where *m* is the mass of the body, *l* is the original leg length, *α* is the angle-of-attack, *y*_*c*_ is the vertical position of the centre of mass, *k* is the stiffness of the leg spring and FP is the foot point of the stance spring. (3) Muscle-reflex (MR) model [23] where point **A** represents the centre of mass of the upper body, *y*_HAT_ is the vertical position of this centre of mass and ***m***_**HAT**_ is the mass of the upper body. For all three models, the vertical unloading force *F*_*u*_ is applied at point **A**. Gait models considered in the paper - filename: figure1.eps

The first goal of this research is to investigate the suitability of gait models for BWS simulation through a comparison with experimentally-obtained gait features. Three prominent biomechanical gait models from the literature are simulated in the sagittal plane with BWS, and trends for gait parameters are documented. The three gait models (Fig. 1), in increasing order of complexity are: (1) *Simplest Walking (SW) model*, (2) *Spring Loaded Inverted Pendulum (SLIP) model* and (3) *Muscle-reflex (MR) model* [21–23].

The second goal is to compare the effect of three fundamental BWS strategies on human gait: (1) *Constant-Force* (CF): which emulates a constant vertical unloading force (2) *Counterweight* (CW): where a vertically moving counterweight is used to provide the unloading force and (3) *Tuned-Spring* (TS): where an elastic element (spring) with specifically tuned stiffness generates the unloading force. The latter two strategies are seemingly ‘imperfect’ realizations to achieve constant vertical support. By comparing their influence on gait parameters to that of an ideal constant unloading force, we aim to explore whether these imperfections are detrimental to the goals of BWS or even provide unexplored benefits for improving BWS design.

In the elastic BWS, the motion of the attachment point affects the deflection of the spring, thus causing variations in unloading force. The Tuned-Spring BWS system is based on the hypothesis [17] that such a variation is desirable and more beneficial than a constant force, because it maintains the dynamic similarity of gait despite unloading. While a constant unloading force partially compensates for the weight of the user, the inertia of the body still affects the dynamics of the gait. We hypothesize that if the unloading force can be tuned to compensate for both the gravitational and inertial forces, gait dynamics will be less modified. According to this hypothesis, and the associated design method presented in [17], the stiffness of the spring used for providing the unloading force can be tuned to compensate for inertial forces of the unloaded mass, thus enabling gait which is more similar to unsupported walking. This works for a periodic (ideally harmonic) movement of the body, and is quite robust to deviations.

Dynamic similarity [24, 25], based on the Froude number, has been previously used for investigating the effect of BWS on gait [26]. However, a recent work [27] suggested the Froude number alone to be inadequate and proposed a new metric called Modela-w. We thus use the change in Modela-w caused by the different BWS conditions to compare the three BWS strategies and test the above-mentioned hypothesis.

Gait parameter trends produced by the simulations are compared with each other and with the human data trends (dataset available at [28]) obtained from an existing systematic review [12]. These trends resulted from the meta-analysis of around fifty existing studies measuring the influence of body weight support on gait parameters. While this meta-analysis presents data for both patients and healthy subjects, only the latter group is considered for comparison in the present study. Results used for benchmarking the gait models and comparing the BWS strategies are presented in “Comparison of gait models” subsection (Fig. 3 and Table 2) and “Comparison of BWS strategies” subsection (Figs. 5, 4, and Table 4) respectively.

**Fig. 2:**
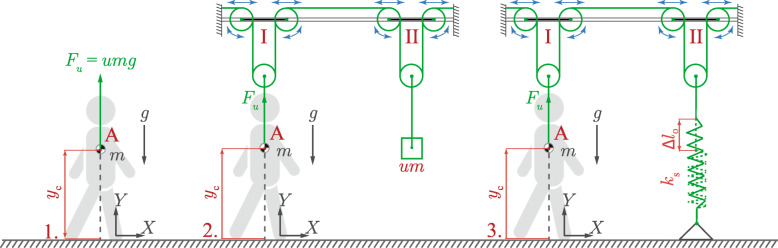
Three BWS strategies: (1) Constant-Force (2) Counterweight system (3) Tuned-Spring system. Pulley systems **I** and **II** are designed such that the counterweight (of mass *u*·*m*) only moves vertically. Also the free end of the spring only moves vertically. The centre of the pulley system **I** is assumed to move horizontally such that force the *F*_*u*_ is directed vertically upwards from point **A**. All pulleys are massless and the system does not dissipate net energy. The coordinate *y*_c_ is the vertical position of the centre of mass of the body, *u* is the amount of body weight unloaded as a proportion of the actual body weight *mg*, *k*_s_ is the stiffness of the spring, and *Δl*_0_ is its initial elongation. BWS strategies considered in the paper - filename: figure2.eps

**Fig. 3:**
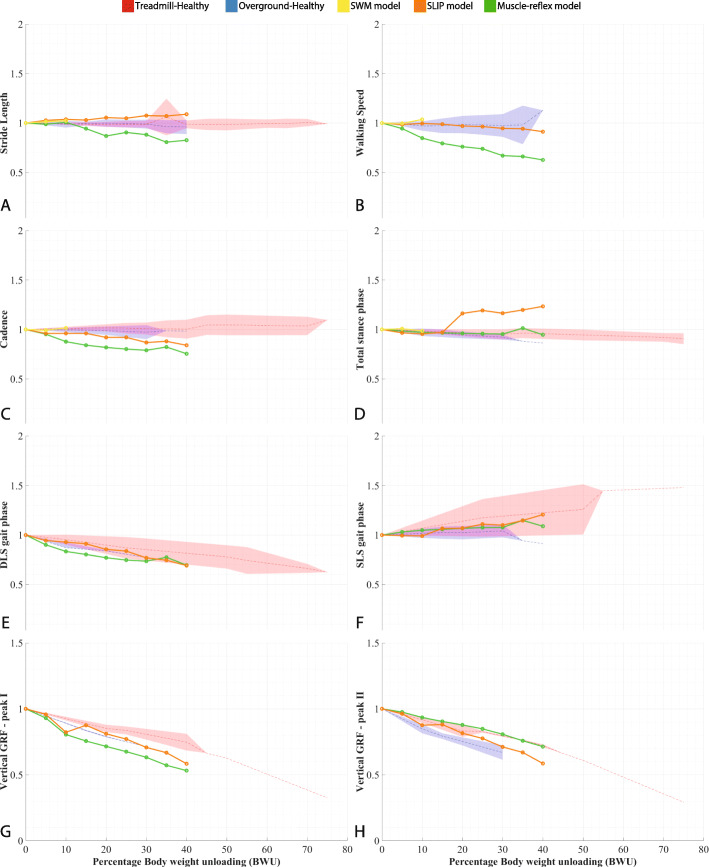
Normalized gait spatio-temporal parameters and vertical ground reaction forces (GRF) where DLS: Double limb support, SLS: Single limb support. Dashed lines represent the mean values and the shaded region represents the standard deviation for human data from [28] Results for the gait characteristics under CF BWS - filename: figure3.eps

**Fig. 4:**
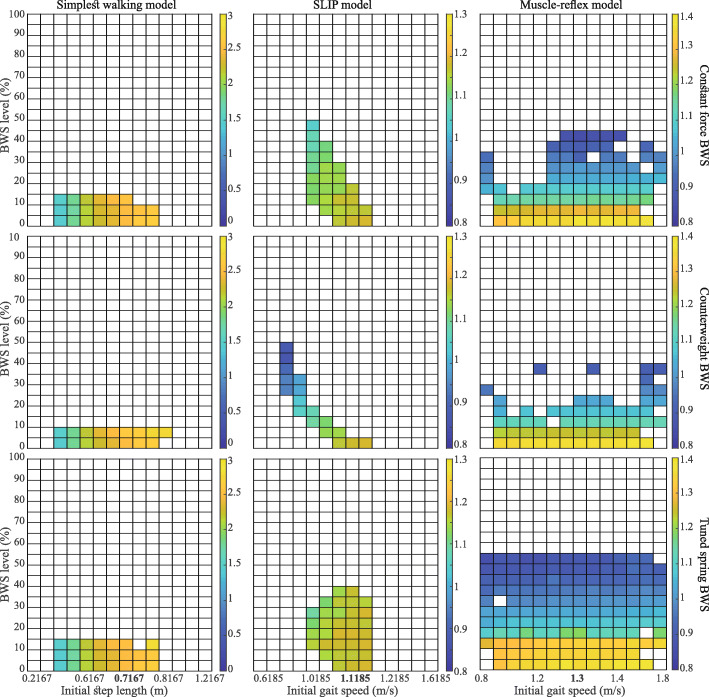
Sensitivity of the average walking speed for each model at different initial walking speeds and BWS levels. The colour bar represents the magnitude of average walking speed over one simulation and the coloured tiles represent the conditions in which the models could walk. The X-axis shows different initial conditions for each simulation; initial step length was varied for Simplest walking model (Column 1), initial gait speed for SLIP and MR models (Columns 2 and 3). Results for the sensitivity analysis - filename: figure4.eps

**Fig. 5:**
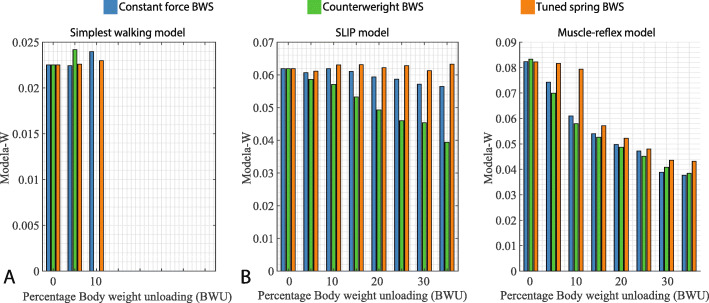
Modela-w values with respect to increasing BWS levels for all three models and BWS strategies. Trends for the Modela-w values - filename: figure5.eps

**Table 1:** Muscle groups in MR gait model and corresponding muscles in experimental data

**MR gait model**	**Experimental data**
1. ‘Vastus’ muscle (VAS) / Quadriceps	Rectus femoris
2. Hamstring (HAM)	Biceps femoris
3. Gastrocnemius (GAS)	Lateral gastrocnemius &
	Medial gastrocnemius
4. Tibialis anterior	Tibialis anterior

**Table 2:** Summary of the gait parameters results for the three gait models

	

Root mean square error (*mrmse*) values for 5–40% Constant-Force BWS levels w.r.t the experimental data for overground (OG) and treadmill (TM) environments are presented here. Lowest *mrmse* values for each gait parameter are indicated in italics and green

## Methods

### Selection of gait models

The scope of this research is limited to 2D gait models since all the gait characteristics of interest, i.e. those studied in [12], can be investigated using 2D models. These characteristics are the gait spatio-temporal parameters and the vertical ground reaction forces; the secondary ones are leg joint range of motion, joint moments, anteroposterior ground reaction forces, and leg muscle activities. These gait parameters are relevant because they have been extensively investigated in previous studies on the influence of BWS and used for designing and testing BWS systems [12]. Four gait models are particularly prominent in literature: (1) Linear inverted pendulum model (LIPM) [29], (2) Simplest walking (SW) model [21] (actuated on the basis of the principles suggested in [30]), (3) Spring-loaded inverted pendulum (SLIP) model [22], and (4) Muscle-reflex (MR) gait model [23]. The LIPM model, however, considers the centre of mass (COM) of the body to move in a straight horizontal line and thus the vertical movement of the COM needed to study the counterweight and tuned-spring BWS strategies is absent. As a result, this model was excluded from the selection of gait models. For the SW model, the foot mass is assumed to be negligible as compared to the body mass. The mechanical configuration and definition of variables for the three models are illustrated in Fig. 1.

### BWS strategies

This section describes the three BWS strategies (Fig. 2) used for simulations, CF, CW and TS.

#### Assumptions

The simulations are based on five main assumptions (Fig. 2) – (1) the counterweight and the free end of the spring only move in the vertical (Y) direction, (2) pulley systems **I** and **II**, the ropes and the spring in Fig. 2 are massless, (3) the BWS system is frictionless and there is no net energy dissipated in the system, (4) the unloading force is applied at the center of mass (COM) of the upper body, which in the cases of SW and SLIP coincides with the body’s overall center of mass, and (5) the pulley system **I** follows the attachment point **A** along the horizontal (X) direction and thus it is always perfectly overhead of the attachment point. This way, the BWS system does not apply any horizontal forces on the gait model nor does it add to the inertia of the model in horizontal direction. While the horizontal force components of the BWS system [31] can be important for determining the user’s gait, we chose to focus solely on the influence of the vertical unloading force on the gait. Considering the % BWS supplied as *β*, the unloading coefficient *u* as *u*=*β*/100, total mass of the body as *m* (Fig. 2) and the acceleration of gravity as *g*, the equations describing the three BWS strategies are presented below.

#### Constant-Force system

The Constant-Force (CF) BWS strategy consists of applying constant vertical force (Eq. 1) on the body. It can be considered as an ideal case of unmodulated BWS [12]. Since the SW and SLIP models do not have distributed mass, the CF BWS strategy also emulates the effects of reduced gravity for these models [14]. However, this is not the case for the MR model [23] due to the presence of limb mass.

1 $$ F_{\text{cf}} = umg  $$

#### Counterweight system

The Counterweight (CW) BWS strategy is based on the use of a counterweight of mass *um* to provide *β* % of BWS. In the static case, this strategy leads to a constant unloading force (*F*_*u*_=*umg*). However, the counterweight moves vertically as it follows the vertical motion of the attachment point **A**. Due to this motion, an additional inertial force ($um\ddot {y_{\mathrm {c}}}$) is generated, which disturbs the intended constant unloading force. Thus, instead of a constant unloading force, the force acting on the body is

2 $$ F_{\text{cw}} = um(g - \ddot{y_{\mathrm{c}}})  $$

where $\ddot {y_{\mathrm {c}}}$ is the vertical acceleration of the attachment point **A** in upward direction.

#### Tuned-Spring system

An elastic element (spring), which can be considered massless as compared to a counterweight, can provide unloading force without the problem of increasing inertial forces caused by the movement of a counterweight. As mentioned above, the spring can even further reduce inertial effects, which in effect means partially removing both gravitational and inertial forces acting on the human body simultaneously [17]. The spring stiffness *k*_s_ to achieve this needs to be tuned to:

3 $$ k_{\mathrm{s}} = u m \omega^{2},  $$

where *ω*=2*πc* and *c* is the cadence (step-to-step frequency) of the walking model at 0% BWS. The initial deflection *Δl*_0_ of the spring is chosen such that the unloading is equal to *umg* in the initial configuration of each model:

4 $$ \Delta l_{0} = \frac{u mg}{k_{\mathrm{s}}} = \frac{g}{\omega^{2}}  $$

The unloading force provided by the TS BWS strategy is:

5 $$ F_{\text{ts}} = k_{\mathrm{s}} (y_{\mathrm{c0}} - y_{\mathrm{c}} + \Delta l_{0}),  $$

where *y*_c_ is the vertical position of point A at time *t* and *y*_c0_ is its average position during walking.

In case of the SW and SLIP models, *y*_c0_ is considered to be the initial position of the model, since the difference between this and the average position is marginal, leading to a small (<3*%*) difference in the intended and actual unloading levels.

For the MR model, choosing the initial vertical position (at *t*=0) of point A as *y*_c0_ leads to higher unloading than desired, whereas the choice of mean vertical position at 0% unloading leads to a lower unloading force. Therefore, for this model, the value of *y*_c0_ was estimated by a heavy first-order low-pass filter on the signal *y*_c_ (Appendix A).

### Model implementation

#### Simulation environments

All gait models were obtained online [32–34] and were modified according to the equations presented below, in order to simulate the effect of all three BWS strategies. For the SW and SLIP models, the equations of motion were implemented in Matlab. In case of the MR model, Simscape blocks were created to emulate the Constant-Force (CF), Counterweight (CW) and Tuned-Spring (TS) BWS strategies, since the original Muscle-reflex gait model [23] was implemented in Simscape.

Only modified equations of motion for each model (Figs. 1 and 2) are presented here. The equations which are not affected by the BWS system are not presented and can be found in the original literature.

#### BWS implementation for the simplest walking model

The original equations of motion of the Simplest walking (SW) model are presented in [21]. The angle *θ* represents the stance leg angle w.r.t. the vertical and *ϕ* is the swing leg angle w.r.t. the stance leg. Following the original paper, time is scaled by $\sqrt {\frac {l}{g}}$ for all three BWS strategies. A hip spring with the dimensionless torsional stiffness *k*_f_ is used for actuation [30]. The ‘foot’ mass *m* is assumed to be much smaller than the ‘body’ mass *M*, so *m*/*M*≈0.

*Constant-Force:* A term representing the constant vertical unloading force (*F*_*u*_=*uMg*) was added to the original equations [21], leading to:

6 $$\begin{array}{@{}rcl@{}} \ddot{\theta} &=& (1-u) \sin \theta \end{array} $$

7 $$\begin{array}{@{}rcl@{}} \ddot{\phi} &=& \ddot{\theta} + \dot{\theta}^{2} \sin \phi + u \sin \theta \cos \phi - \cos \theta \sin \phi - k_{\mathrm{f}} \phi. \end{array} $$

*Counterweight system:* The mass of the counterweight is *uM*, where *M* is the mass at the hip, so

8 $$\begin{array}{@{}rcl@{}} \ddot{\theta} &=& \frac{1-u}{1+u} \sin \theta \end{array} $$

9 $$\begin{array}{@{}rcl@{}} \ddot{\phi} &=& \ddot{\theta} + \dot{\theta}^{2} \sin \phi - \cos \theta \sin \phi \end{array} $$

10 $$\begin{array}{@{}rcl@{}} && + \frac{2u}{1+u} \sin \theta \cos \phi - k_{\mathrm{f}} \phi. \end{array} $$

*Tuned-Spring system:* Considering Eqs. (1-3) in “Methods” section, *y*_c_=*l* cos*θ* to be the vertical position of point A at time *t* and *y*_c0_=*l* at *t*=0, the EOM are:

11 $$\begin{array}{@{}rcl@{}} \ddot{\theta} &=& (1-u)\sin \theta + \frac{l}{g} \omega^{2} u (1- \cos \theta) \sin \theta \\ \ddot{\phi} &=& \ddot{\theta} + \dot{\theta}^{2} \sin \phi - \cos \theta \sin \phi  \end{array} $$

12 $$\begin{array}{@{}rcl@{}} && - u (1 + \frac{l}{g} \omega^{2} (1 - \cos \theta)) \sin \theta \cos \phi - k_{\mathrm{f}} \phi. \end{array} $$

#### BWS implementation for the bipedal spring-loaded inverted pendulum model

The gait cycle in the SLIP model given in the original paper [22] is divided into three phases – initial single limb stance (SLS) of the left leg, intermittent double-limb stance (DLS) and final single limb stance (SLS) of the right cycle. The equations for horizontal acceleration do not change since BWS is assumed to influence only the vertical motion. The modified equations of motion for the vertical motion of the COM depend on the chosen BWS strategy.

*Constant-Force system:* A term representing the constant vertical unloading force (*F*_*u*_=*uMg*) is added to the original equations [22], so:

13 $$\begin{array}{@{}rcl@{}} \text{Initial SLS:} \quad m \ddot{y}_{\mathrm{c}} &=& P y_{\mathrm{c}} - m(1-u)g. \end{array} $$

14 $$\begin{array}{@{}rcl@{}} \text{ DLS:} \quad m \ddot{y}_{\mathrm{c}} &=& P y_{\mathrm{c}} + Q y_{\mathrm{c}} - m(1-u)g. \end{array} $$

15 $$\begin{array}{@{}rcl@{}} \text{Final SLS:} \quad m \ddot{y}_{\mathrm{c}} &=& Q y_{\mathrm{c}} - m(1-u)g. \end{array} $$

*Counterweight system:* The mass of the counterweight is *um*, where *m* is the mass of the body, thus leading to:

16 $$\begin{array}{@{}rcl@{}} \text{Initial SLS:} \quad m \ddot{y}_{\mathrm{c}} &=& P y_{\mathrm{c}} - m\frac{(1-u)}{1+u}g. \end{array} $$

17 $$\begin{array}{@{}rcl@{}} \text{ DLS:} \quad m \ddot{y}_{\mathrm{c}} &=& P y_{\mathrm{c}} + Q y_{\mathrm{c}} - m\frac{(1-u)}{1+u}g. \end{array} $$

18 $$\begin{array}{@{}rcl@{}} \text{Final SLS:} \quad m \ddot{y}_{\mathrm{c}} &=& Q y_{\mathrm{c}} - m\frac{(1-u)}{1+u}g. \end{array} $$

*Tuned-Spring system:* Considering Eqs. (1, 2 and 3) in “Methods” section, the resulting equations for the Tuned-Spring strategy are:

19 $$\begin{array}{@{}rcl@{}} \text{Initial SLS:} \quad m \ddot{y}_{\mathrm{c}} &=& P y_{\mathrm{c}} - mg + F_{\text{ts}}. \end{array} $$

20 $$\begin{array}{@{}rcl@{}} \text{ DLS:} \quad m \ddot{y}_{\mathrm{c}} &=& P y_{\mathrm{c}} + Q y_{\mathrm{c}} - mg + F_{\text{ts}}. \end{array} $$

21 $$\begin{array}{@{}rcl@{}} \text{Final SLS:} \quad m \ddot{y}_{\mathrm{c}} &=& Q y_{\mathrm{c}} - mg + F_{\text{ts}}, \end{array} $$

where *F*_ts_ is the unloading force provided by the TS BWS system (equation 3), *y*_*c*_ represents the vertical position of the COM of the body. The terms *P* and *Q* are the same as those defined in [22]

$$ P = k(\frac{l_{0}}{\sqrt{x_{c}^{2}+y_{\mathrm{c}}^{2}}} - 1) \quad \& \quad Q= k(\frac{l_{0}}{\sqrt{(d-x_{c})^{2}+y_{\mathrm{c}}^{2}}} - 1), $$

where $ d = \text {FP}_{i+1,x_{c}} - \text {FP}_{i,x_{c}} $, FP is the foot point of the stance spring, and *x*_*c*_ is the horizontal position of the COM.

#### BWS implementation for the muscle-reflex model

The unloading force term for each BWS strategy were implemented as Simscape blocks according to the equations in section IIB, where Eqs. 1, 2 and 5 presents the unloading force for the CF, CW and TS BWS strategies respectively.

### Simulation protocol

Each modified model was simulated with BWS ranging from 0% to 100%, in 5% increments. The unloading force was applied at the center of mass of the body (COM_body_) for all gait models and the COM of the upper body for the MR model (Appendix B). Initial pose for the SLIP and MR models is standing, at the instant before toe-off, while it is in the double stance after impact in case of the SW model. To test the model sensitivity to initial conditions, fifteen different starting gait speeds were used for simulation, ranging from 0.6185 m/s to 1.6185 m/s for the SLIP model (original 1.1185 m/s [22]) and 0.8 m/s to 1.8 m/s for the MR model (original 1.3 m/s [23]). Since the starting gait speed is not selectable for the SW model, starting step length was varied from 0.2167 to 1.2167 m (original 0.7167 m [21]). The remaining initial conditions and model parameters used in simulation are the same as those proposed in the original papers [21–23]. The highest percentage of BWS for which the model was able to achieve a walking gait for at least 20 steps was noted as the ‘Maximum feasible BWS’ (*β*_max_) for each strategy. This *β*_max_ and the resultant average gait speed were used to understand the sensitivity of gait models to initial conditions.

Results for CF BWS strategy were selected for the comparison with experimental data since this data [28] was available only for Constant-Force BWS systems [12].

### Data analysis

#### Selection and analysis of gait parameters

Relevant gait data was extracted for the starting gait speed or step length which was closest to the values proposed in the original papers and which led to the highest maximum feasible BWS. For each condition, the gait data was averaged over at least five strides in order to reduce the variability. The average step duration was considered as the inverse of cadence.

We also calculated the proportion of each gait phase with respect to the entire stride duration. The hip range of motion was calculated from the peak flexion angle following initial contact to the peak extension angle at terminal stance [35]. The knee range of motion was considered from the peak extension angle at terminal stance to the peak flexion angle at mid-swing. Peak joint torque values for flexion and extension were extracted from the torque patterns over a complete gait cycle, and indicated by negative and positive signs, respectively. The two peak values for the vertical ground reaction forces (GRF) and the extrema of the anteroposterior GRF over a single gait cycle were also calculated. For muscle activity, the mean value over a complete gait cycle was considered. Some model-specific data analysis procedures were adopted, listed below:

- The SW model was analyzed only for the gait spatio-temporal parameters like stride length, cadence, walking speed and the total stance phase. The model has an instantaneous double support phase, so only the total stance phase is considered.
  Ground reaction forces (GRF) were not considered either, since they do not follow the characteristic pattern of anthropomorphic bipedal gait [36].
- The SLIP gait model was investigated for all gait spatio-temporal parameters and the vertical GRF.
- Since the MR gait model typically utilizes muscle groups, while the meta-analysis of experimental gait data [12] provides muscle activity data for individual muscles, the correspondence in Table 1 was used for comparing the results.

#### Comparison of gait parameter response

Suppose $\phantom {\dot {i}\!}P = [P_{0},P_{5},P_{10},....,P_{\beta _{\text {max}}}]$ represents the vector of values for a specific gait parameter at each unloading level up to ‘Maximum feasible BWS’ (*β*_max_), in increments of 5%. This data was normalized by taking a ratio with the parameter value at 0% BWS, resulting in $\phantom {\dot {i}\!}P_{N} = P/P_{0}= [1,P_{5}/P_{0},P_{10}/P_{0},....,P_{\beta _{\text {max}}}/P_{0}]$ The aim was to reduce variability in results and allow comparison of trends across gait models. By removing the dimensions attached to each parameter through normalizing, comparison across different gait parameters was possible. The data from the meta-analysis [12] was used as reference human data, *P*_*H*_=[1,*P*_*H*5_,*P*_*H*10_,....,*P*_*k*_]. Because this reference data is already normalized, the normalization procedure for the gait model parameters enabled comparison with human data.

To compare the gait models, for each gait parameter, the root mean square error with respect to the experimental data was calculated for overground and treadmill walking environments. Since it is used to compare the gait models, this root mean square error is referred to as mrmse. The mrmse (Eq. 22) was computed as a percentage of the gait parameter value at 0% BWS. The 0% BWS condition was not considered during mrmse calculation since the gait parameter data was normalized, such that the error at 0% BWS was always 0. A lower value of the mrmse, so a better fit with the experimental data, means that the model is better suited to investigate the influence of BWS on that specific gait parameter. The comparison of gait models is based only on the mrmse values for the overground condition with a Constant-Force BWS system. The data considered for analysis ranged from 0% to 40% BWS, because at least two models could not achieve stable gait above 40%. A missing data point indicates inability of the gait model and BWS strategy to produce a gait at that BWS level, which was penalized during the calculation of mrmse to reflect higher error values. *P*_*N*_ was considered to be zero for this BWU level, since this can be considered as the highest deviation from the gait at 0% and thus it leads to the largest possible error for that unloading level. Human data indicates only a small influence of unloading force on gait parameters up to 30% BWS [12], which implies that the mrmse w.r.t. to the 0% BWS should also be small for the gait models, up to 30% unloading:

22 $$ \text{mrmse} = \sqrt{\frac{\sum_{k = 1}^{8}(P_{H}(k)-P_{N}(k))^{2}}{3}} \times 100\%,  $$

where k is the index of the BWS levels, which are *k*·5*%*, thus ranging from 5 to 40%.

In the context of BWS, the level of dynamic similarity between the unloaded walking task and the eventual task to be trained, namely walking without support. In order to quantify this dynamic similarity, we first represent the gait dynamics for each condition by a dimensionless number, modela-w [27]:

23 $$ \text{modela-w} = (\frac{2gl}{v^{2}} + (\frac{f l}{v})^{2})^{-1},   $$

In the next step, we quantify the loss in dynamic similarity by calculating the combined root mean square difference of the modela-w magnitude from 5% to 35% unloading with respect to the magnitude at 0% BWS for the SLIP and MR models For the SW model, it was calculated only from 5% to 10% unloading for CF and TS strategies, whereas only at 5% for the CW strategy. To compare BWS strategies, we assume that those which lead to lower root mean square difference values are likely to distort gait dynamics less. This root mean square error is referred to as *Δ*gd (Eq. 24) because it forms the basis for comparison of change in gait dynamics or the loss of dynamic similarity:

24 $$ \Delta \text{gd} = \sqrt{\frac{\sum_{k = 1}^{7}(\text{modela-w}(n)-\text{modela-w}(0))^{2}}{7}} \times 100\%,   $$

where *k* represents the BWS levels in the form of *k*·5*%*, ranging from 5% to 35%.

## Results

### Comparison of gait models

**Fig. 6:**
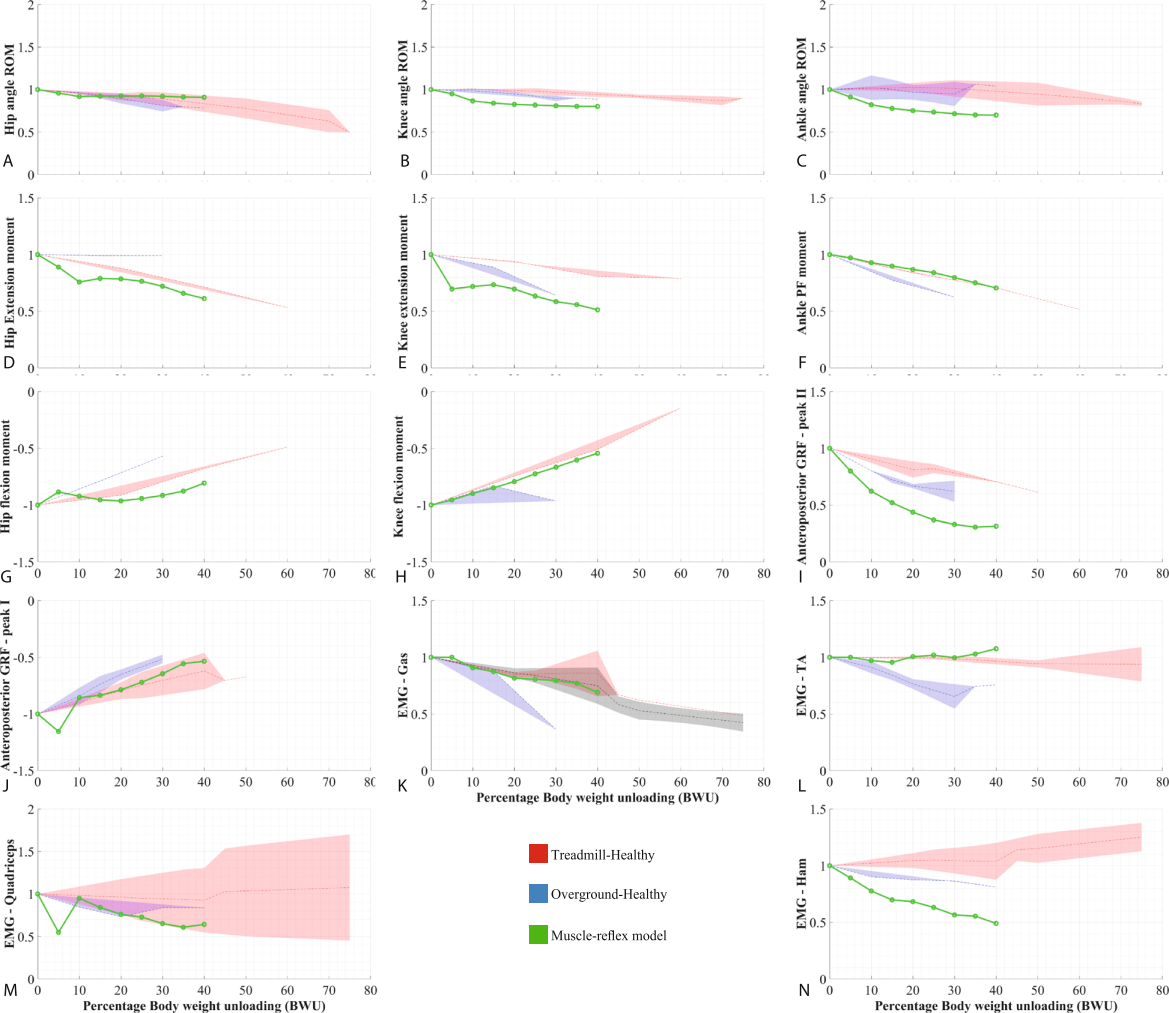
Gait dynamics where ROM: Range of motion, PF: plantarflexion, GRF: ground reaction forces and AP: anteroposterior. Dashed lines represent the mean values and the shaded region represents the standard deviation for human experimental data. Gait dynamics - filename: figure6.eps

The gait parameter values at different levels of Constant-Force BWS for each gait model are plotted in Fig. 3, along with the experimental data obtained from the meta-analysis [12] for healthy individuals walking in overground and treadmill environments. Gait parameters which were present only in one model, i.e the MR model, are included in Appendix C (Fig. 6). The *mrmse* for each model and the relevant gait parameters are presented in Table 2. Values for the treadmill condition are presented only for comparison with the overground condition for the same model and not between two models. Results for the sensitivity analysis are shown in Fig. 4.

#### Simplest walking model

The SW model had the highest *mrmse* values for all relevant gait parameters: stride length, cadence, walking speed, and total stance phase (Fig. 3 & Table 2). The magnitude of *mrmse* was similar for overground and treadmill walking. Average gait speed showed low sensitivity (Fig. 4) to the magnitude of BWS but was relatively high for the initial step length; the sensitivity hardly changed for different BWS strategies.

#### Spring-loaded inverted pendulum gait model

The SLIP model had the lowest *mrmse* values for six out of eight paramaters, including stride length, cadence, walking speed, double limb stance, and vertical GRF (Fig. 3). For single limb stance phases, it showed a moderate *mrmse* value, comparable to the MR gait model, while for total stance phase, it was almost twice that of the MR model. Apart from the cadence, double limb stance, and vertical GRF peak I, the four remaining gait parameters had a higher *mrmse* value for overground walking. Average gait speed for SLIP model was highly sensitive (Fig. 4) to the initial speed for the CW BWS strategy while being relatively low for the other two strategies; it was low with respect to the level of BWS for all three BWS strategies.

#### Muscle-reflex gait model

The Muscle-reflex (MR) gait model was the only one which could be tested for almost all gait parameters mentioned in the meta-analysis [12]. Of the 23 gait parameters analyzed, this model had a *mrmse* of less than 10% for only eight characteristics (Table 5): total stance phase, double limb stance, vertical GRF peak I, hip joint ROM, knee flexion moment, ankle plantarflexion moment, gastrocnemius, and tibialis muscle activity. Except for single limb stance, and total stance phases, the MR model had higher *mrmse* values for other gait parameters than the SLIP model but lower than the SW model (Table 2).

However, the MR model had a high *mrmse* for knee and hip extension moments, anteroposterior GRF, ankle joint ROM and quadriceps muscle activity (Appendix C - Fig. 6). Furthermore, for 8 gait parameters, the MR model showed lower *mrmse* values for the treadmill walking condition than for the overground condition. These parameters included total stance, single limb stance, vertical GRF peak II, hip moments, ankle plantarflexion moment, anteroposterior GRF peak I and tibialis anterior muscle activity (Fig. 3, (Table 5), and Appendix C - Fig. 6). Sensitivity of the average gait speed (Fig. 4) was low towards the initial speed and high for the level of BWS, for all three BWS strategies.

### Comparison of BWS strategies

The highest feasible BWS (*β*_*max*_) values for the three models and the BWS strategies are presented in Table 4. The trends for modela-w values are plotted in Fig. 5 and the range of feasible BWS levels across different initial gait speeds and step lengths (SW model) are plotted in Fig. 4. The range here refers to the difference between the minimum (not necessarily 0%) and maximum feasible BWS levels. The change in gait dynamics (*Δ*gd) or the loss of dynamic similarity for each model in each BWS strategy is presented in Table 3.

**Table 3:** Maximum value of BWS (*β*_*max*_) at which the model still achieved a walking gait

	**Constant-Force**	**Counterweight**	**Tuned-Spring**
*SW model*	10	5	10
*SLIP model*	45	45	35
*MR model*	40	35	50

**Table 4:** Change in the dimensionless constant *Modela-w* for all models and under each BWS strategy

	

Root mean square of the difference between the magnitude of Modela-w from 5% to 35% BWS levels with respect to the magnitude at 0% BWS is shown here. This metric (*Δ*gd) is calculated as percentage and the least change in Modela-w i.e. the highest dynamic similarity condition is indicated in green and italics

**Table 5:** Summary of the results of gait parameters for the MR model

**Root mean square error (*m-rmse*) values**						
**Experimental data**	**Overground (OG)**			**Treadmill (TM)**		
**Gait model**	**SW**	**SLIP**	**MR**	**SW**	**SLIP**	**MR**
**Gait parameter**	%	%	%	%	%	%
01. Hip joint - ROM	–	–	2.84	–	–	2.83
02. Knee joint - ROM	–	–	10.36	–	–	12.51
03. Ankle joint - ROM	–	–	18.27	–	–	18.61
04. Hip extension moment	–	–	18.85	–	–	13.46
05. Hip flexion moment	–	–	10.60	–	–	5.88
06. Knee extension moment	–	–	21.49	–	–	25.47
07. Knee flexion moment	–	–	1.32	–	–	2.93
08. Ankle plantarflexion moment	–	–	9.00	–	–	1.35
09. Anteroposterior GRF peak - I	–	–	14.60	–	–	12.14
10. Anteroposterior GRF peak - II	–	–	16.67	–	–	27.06
11. Muscle activity - Quadriceps	–	–	22.60	–	–	26.73
12. Muscle activity - Hamstrings	–	–	13.53	–	–	24.99
13. Muscle activity - Medial Gastrocnemius	–	–	–	–	–	2.79
14. Muscle activity - Lateral Gastrocnemius	–	–	2.47	–	–	2.52
15. Muscle activity - Tibialis anterior	–	–	7.84	–	–	2.84

Root mean square error (*m-rmse*) values with respect to the experimental data for overground (OG) and treadmill (TM) environments are presented here

#### Counterweight BWS

The Counterweight (CW) BWS strategy typically led to the lowest *β*_*max*_ values (Table 3) for the SW and MR models, and lowest feasible BWS ranges (Fig. 4) across all three gait models. This is reflected in the high *Δ*gd values (Table 4) for all models and a stronger change in modela-w values (Fig. 5) for the SW and SLIP models. Sensitivity of the *β*_*max*_ to initial conditions was highest in case of CW BWS strategy (Fig. 4), especially for the SLIP model.

#### Constant-force BWS

With regards to the SW and MR gait models, the constant-force (CF) BWS strategy typically produced a higher *β*_*max*_ value than CW BWS and a lower value than tuned-spring (TS) BWS (Table 3), and vice-versa for *Δ*gd values (Table 4) for all three models. It showed a similar trend for the range of feasible BWS levels (Fig. 4) and the modela-w values(Fig. 5). Finally, the sensitivity of *β*_*max*_ to initial conditions was lower than the CW strategy and higher than TS.

#### Tuned-Spring BWS

The Tuned-Spring (TS) produced the highest *β*_*max*_ values among all three BWS strategies for SW and MR models, while leading to high but not the highest values for the SLIP model. It also led to lowest change in gait dynamics (Table 4 and Fig. 5) across all models. Further, the *β*_*max*_ values were least sensitive to the initial condition (Fig. 4) for the TS BWS strategy in case of the SLIP and MR models.

## Discussion

### Comparison of gait models

The SW model showed the highest mrmse values for all four gait parameters (Table 2), namely stride length, cadence, walking speed, and stance phase duration. While Fig. 3 does not reflect such high mrmse values (≈50%), these values are expected due to the penalization process explained in the earlier “Comparison of gait parameter response” section. Onwards from 15% BWS, the SLIP model presented a sudden increase in the proportion of single limb stance phase and consequently for total stance phase, relative to human data and the MR gait model, which led to a high *m-rmse*. This phenomenon can be attributed to the stabilization effect of the unloading force during the single limb stance. This effect was more pronounced in the SLIP model than in the MR model, as the MR model is comparatively more robust to disturbances [23]. However, for other parameters, the SLIP model showed the best performance out of the three models, despite its relative simplicity. Typically, the aim of active BWS is to enable the magnitude of spatio-temporal parameters to be similar to the values during unsupported walking or to retain the M-shape of the vertical GRF [37, 38]. These gait parameters are present in the SLIP model, and they change in similar ways as in the experimental data. This indicates that the SLIP model can likely be used effectively to simulate the effects of modulated BWS on gait spatio-temporal parameters and ground-contact interactions.

While the SW and MR models add energy to the system to maintain stable walking, the SLIP model does not. This feature of the SLIP model might be one of the reasons for its good performance and is worthy of further investigation. The SLIP model was only tested up to 40% BWS since the MR model could not achieve a walking gait beyond that level and thus no data was available for comparison of models. The accuracy of the SLIP model for higher BWS levels could not be investigated.

In case of the MR model, the unloading force produced an additional torque about the hip joint which needed to be counter-balanced by muscle forces. This led to an increase in the hip flexion moment and a decrease in the hip extension moment and subsequently affected the knee extension moment as well (Fig. 6). While analyzing the data, it was noted that the peak knee extension torque shifted temporally from just after initial contact to just before toe-off at 10% BWS. This temporal change in torque peak led to a sharp drop in knee extension moment magnitude, as seen in Fig. 6E. This could explain the sizable deviations from the human data for the hip and knee joint moments and thus the high mrmse (Table 5). While the ankle plantarflexion moment in the MR model was less affected by BWS than in humans, ankle angle ROM dropped almost 20% lower than the human data. This reduction in ankle ROM, in combination with lack of change in ankle plantarflexion moment, led to a higher reduction in the forward push-off force (anteroposterior GRF peak II - Fig. 6I) and a lower reduction in the vertical push-off force (vertical GRF peak II – Fig. 3H) as compared to human data. In case of the muscle activities, muscle groups in the MR gait model were compared to individual muscles in the experimental data (Table 1). While the muscle activities of the individual muscles are correlated to the muscle groups [39], the MR model showed high mrmse values (>10*%*) for all muscles (Table 5) except the Lateral Gastrocnemius (LG) muscle.

The muscle reflexes and initial conditions for the MR model were not optimized for BWS, which might partially explain its lower performance. While an optimization would likely have led to a higher value of *β*_*max*_, the non-optimized model still yields comparatively high *β*_*max*_ values (Table 3). However, hand-tuning the model to suit every modulated BWS level would require extensive human data from experiments with modulated BWS, and obtaining this data is difficult. While optimization algorithms can be used to tune the model parameters [40], designing an appropriate cost function is difficult. Yet, this model could still be useful in certain scenarios, wherein the muscle reflexes could be tuned to emulate the pathological muscle function in individuals with neuromuscular disorders. Further, the MR model can also be used to optimize the body weight support training for biomechanical outcomes such as joint loading, investing the impact of different BWS attachment points on the upper body, etc. Finally, the *β*_*max*_ values for the MR model are less sensitive to the initial gait speed than the SLIP model. Thus, the MR model offers a more robust alternative to the SLIP model for simulating a wider variety of initial conditions, albeit with a lower accuracy.

The starting conditions in the simulation for each model were selected based on their ability to produce the maximum feasible body weight support level. While this leads to the comparison of models under differing simulation conditions, the evaluation concerned only the ability of the models to reproduce the gait parameter trends in response to different levels of BWS, but not the ability of the models to reproduce gait without BWS. Therefore, there remains a bias absolute error for all models. The absolute errors have been evaluated in [21–23]. Further, the maximum feasible BWS support for the SLIP and MR models is sensitive to the initial gait speed for the CF BWS condition, which makes it pertinent to select the appropriate initial speed.

### Performance of the BWS strategies

The three BWS strategies evoked very different responses, especially in the SLIP and MR models. The *β*_*max*_ values were typically highest for the TS BWS strategy and lowest for the CW BWS strategy (Table 3). This highlights the importance of considering inertia in the design of BWS systems. Fig. 5 shows that TS BWS had a lower influence on gait dynamics than the other two strategies. It also led to a more consistent range (Fig. 4) of feasible BWS across all initial conditions. In case of SLIP and MR models, the TS BWS produced the lowest *Δ*gd values for all gait models (Table 4).

High *β*_max_ and low *Δ*gd values for the TS BWS strategy support the hypothesis that a spring-based BWS can enable gait which is more similar to unsupported walking [17]. Ideally, dynamic similarity in this case means that also the neural control strategy does not need to be changed when walking under the influence of body weight support. This makes a case for experimental evaluation, and questions the predominant paradigm of perfectly controlled constant unloading forces as being preferable to simple elastic support. Unloading force rendered by the TS BWS depends on the initial zero-deflection set-point of the spring and thus, it is important to consider an appropriate value for the initial set-point during the experimental evaluation of this strategy. Active BWS systems like the ZeroG [13], the FLOAT [41], the RYSEN [42], etc. could measure and slowly adjust to the average position during walking in real time. For simpler or passive BWS systems, using the standing position to adjust unloading force is the most practical option, although it may lead to a bias in the average unloading force.

### Viability of using simple gait models

The model-predicted outcomes such as gait spatio-temporal parameters and ground reaction forces followed similar qualitative trends (increasing/decreasing) as the human data, despite high mrmse values for some parameters like the joint moments and muscle activities. This indicates that the response of gait models to BWS is akin to that of humans, albeit slightly exaggerated. The three gait models showed a stronger influence of BWS on most gait parameters than the experimental human data for both treadmill and overground walking conditions (Fig. 3 & Appendix C - Fig. 6). While the human data presented a higher influence of BWS on the kinetic gait characteristics than on the gait spatio-temporal parameters and joint angles, the gait models also presented a larger effect for knee and ankle joint angle ROM, cadence, walking speed and double limb support phases. This was reflected in the higher mrmse for the cadence, walking speed and the joint angle ROM, as compared to the mrmse values for other spatio-temporal parameters, especially for the MR model.

In case of the CF BWS strategy, the range of *β*_*max*_ values lied between 40% to 45% for the SLIP and MR gait models (Table 3). Above this range, the unloading force led to such a strong influence on the gait parameters that the gait models were unable to attain a walking gait. This range of *β*_*max*_ values aligns to the 30% BWS level, up to which the influence of BWS on gait spatio-temporal and kinematic parameters has been shown to be limited [2, 12, 43–47].

For simulating higher BWS levels, it could be useful to refer to studies by Glauser et al. [18] and Ma et al. [20] which employ simulation techniques to predict the influence of BWS on human gait. The first study [18] employs a mass-spring-damper (MSD) system with two lumped masses representing the upper and the lower body, while the second study uses human motion capture data in conjunction with LifeMO*D*^*TM*^ simulation package. However, it is necessary to note that the MSD model has not been validated against human data, while the second method is cumbersome and can be biased by the subject-specific nature of the motion capture data.

### Limitations of this study and potential directions for further research

A major limitation of this study is that it only considers movement in the sagittal plane. It has been shown that BWS also impacts gait, particularly balance, in other planes [48, 49].

A possible extension of this study would be to consider other models suggested for gait, for example based on optimization [50, 51], neural control and central pattern generators [52–54], the Virtual Pivot Point (VPP) [55], or the capture point [56]. Nonetheless, the selected gait models already cover most of the main features of human gait like mechanical stability, compliant nature of legs, segmented legs, muscle-reflex architecture, and the m-shape of vertical GRF [57].

Cost of transport (COT) or metabolic cost for walking could be another measure to analyze when comparing gait models. It is known that COT decreases with the increase in BWS and that COT is an important governing factor for gait transitions [12]. Mechanical work could be calculated from the joint power consumption. However, while this work is correlated to the COT, it cannot be used to accurately determine the COT [58].

The point of application of the unloading force on the upper body may also play an important role. For the MR model, the *β*_*max*_ values for the TS and CF BWS strategies were highest if the unloading force was applied close to the COM of the upper body (Appendix B). This suggests that any moment of the unloading force on the upper body, even if the force is applied at a small distance from the upper-body COM, has a destabilizing effect. An in-depth investigation of the behavior of gait characteristics for different locations of the BWS application point could be useful for the design of harness systems and for choosing between pelvic or body harness-based attachments. In case of the CW BWS strategy, the effective COM location changes due to the counterweight, thus making it difficult to predict the *β*_*max*_ behaviour.

In contrast to the predominant goal of constant, or *unmodulated* unloading force in active BWS, also *modulated* active BWS has been suggested, where the unloading force is controlled according to specific gait parameters. Recently, some interesting modulated BWS systems have been suggested [12], such as one that controls the unloading force based on gait cycle phases [37], another where the centre of pressure trajectory governs the unloading force [59], and a system that aims to dynamically compensate the inertial forces of the user’s body [38]. It appears that modulation of unloading force can facilitate appropriate ground contact and limb motion while allowing gait spatio-temporal parameters like walking speed, cadence and stride length to remain comparable to the values during unsupported walking [12]. Evidence still remains limited to pilot studies though.

Several BWS designs also allow for modulation of the force vector in other directions than the vertical [13, 41, 42]. Appropriate interplay of vertical and forward forces may be another mechanism when striving for similar gait dynamics [31]. Simulation of gait models with modulated vertical and/or forward forces can provide the first step towards the detailed experimental studies for validating modulated BWS designs.

## Conclusion

The primary goal of this research was to benchmark widely used gait models based on their suitability to the simulation of human walking with body weight support. Gait models were simulated under the influence of Constant-Force, Counterweight, and Tuned-Spring BWS strategies. The results of this work strengthen the idea that reasonably simple gait models can be effectively used to simulate the effects of body weight unloading on human locomotion. This study demonstrates the usefulness of gait models for BWS simulation, with the SLIP model having matched the human data more closely than the Simplest Walker and the Muscle-reflex models. However, the viability of gait models varies strongly with the type of BWS strategy and the initial gait speed. The results also point to limitations of the widely-used models in responding in a realistic way to external forces, indicating that they should be used only with caution outside of the situations they were developed, tuned, and evaluated for. Furthermore, the simulation results for the Tuned spring BWS strategy show promise and merit experimental investigation to compare its influence on human gait with that of a closed-loop control-based constant unloading strategy.

## Appendix A: Implementation of TS BWS strategy

The Tuned-Spring (TS) BWS strategy is implemented using a first-order low-pass filter which is used to obtain the value of reference position of the spring, indicated by *y*_*c*0_ in Eq. 3. The rise time of this filter, from 0 to the value of the mean vertical position of the COM, is roughly 15 seconds. During initialization, the filter output is compared to the average vertical COM position at 0% BWS (1.3037 m) using an IF block, and the higher value among these two is used as the reference position *y*_*c*0_.

## Appendix B: Force application point for the MR model

For the MR model, the selection of the location where the unloading force acts is an important decision. Since the limbs in this model are assumed to have mass, the center of mass of the body (CO*M*_*body*_) is different from the center of the mass of the upper body which includes the head, arms and trunk (CO*M*_*HAT*_) and excludes the legs. The distance of the CO*M*_*body*_ from the hip joint (*d*), along the length of the upper body, was calculated using the CO*M*_*body*_ position at three initial symmetric standing configurations: (1) legs at 90^*o*^ to horizontal (2) legs at 45^*o*^ to horizontal and (3) legs at 0^*o*^ to horizontal, a fictitious boundary case. The parameter *d* was highest in the third case (0.2341m) and so the *β*_*max*_ was computed at *d* ranging from 0.23m to 0.7m, 0.7m being two times the distance of CO*M*_*body*_ from hip joint. The magnitude of *β*_*max*_ is highest typically around the position of the CO*M*_*HAT*_ for CF and TS strategies, while it did not show a consistent behaviour for the CW strategy. Thus, the CO*M*_*HAT*_ was chosen as the point of application of the unloading force since it is a well-defined point and leads to high *β*_*max*_ values.

## Appendix C: Additional results for gait parameters

Additional parameter trends are presented in Fig. 6. These only concern the MR model and hence do not allow comparison between the gait models. Joint dynamics, anteroposterior GRF and muscle activity plots are included here.

## Abbreviations

BWS: Body weight support
SW: Simplest walking (gait model)
SLIP: Spring-loaded inverted pendulum (gait model)
MR: Muscle-reflex (gait model)
ROM: Range of motion
GRF: Ground reaction forces
DLS: Double limb stance phase
SLS: Single limb stance phase
